# The potential of CD127 as a prognostic and residual disease marker in chronic adult T cell leukaemia/lymphoma

**DOI:** 10.1186/1742-4690-11-S1-P9

**Published:** 2014-01-07

**Authors:** Huseini Kagdi, Anat Melamed, Silva Hilburn, Nicolas Gillet, Andrew Hodson, Maria A Demontis, Aileen Rowan, Lucy Cook, Charles Bangham, Graham Taylor

**Affiliations:** 1 Infectious Diseases and Immunology, Faculty of Medicine, Imperial College, London, UK; 2 Molecular and Cellularepigenetics, Interdisciplinary Cluster for Applied Genoproteomics (GIGA) of University of Liège , Leige, Belgium

## 

Adult T cell Leukaemia Lymphoma [ATL], a mature T-cell neoplasm has been classified into 4 subtypes: smouldering; chronic leukaemia; lymphoma and acute leukaemia. The diagnosis depends on clinical features, the immunophenotype, and demonstration of HTLV-1 infection & ideally of monoclonal proviral integration. The typical immunophenotype of ATL is not specific. The methods used to detect monoclonality are labour-intensive and/or expensive and are not widely available. We developed a flow cytometry assay for diagnosis and monitoring of ATL. We performed 11-colour immunophenotyping , HTLV-1 proviral quantification and proviral integration site [IS] analysis on 53 samples from 36 patients [25 non ATL HTLV-1-infected,11 chronic/smouldering ATL], 3 uninfected individuals and 2 HTLV-1-immortalized cell lines. The non-ATL patients had CD127+ & CCR7-lo expression in CD4+CD25+CCR4+ cells, and a polyclonal distribution on IS analysis. Four ATL patients had CD127+ & CCR7-lo expression in CD4+CD25+CCR4+ cells and polyclonal distribution on IS analysis. These patients had an excellent outcome achieving remission with either PUVA or no therapy. Eight ATL patients had CD127-lo expression on CD4+ CD25+ CCR4+ cells with mono/oligoclonal distribution on IS analysis. One of nine patients with chronic ATLL had high CCR7 expression. Foxp3 expression was variable. All 8 patients required systemic ATL treatment and longitudinal study of 5 patients found the change in frequency of CD4+CD25+CCR4+ to correlate with PVL whilst CD127 expression correlated with IS analysis (p<0.005) and disease remission status. CD127 expression appears to be useful to identify patients needing treatment and for monitoring the treatment of chronic ATL.

